# FKBP25 participates in DNA double-strand break repair^1^

**DOI:** 10.1139/bcb-2018-0328

**Published:** 2019-01-08

**Authors:** David Dilworth, Fade Gong, Kyle Miller, Christopher J. Nelson

**Affiliations:** The Department of Biochemistry & Microbiology, University of Victoria, Victoria, BC V8W 3P6, Canada.; Institute for Cellular and Molecular Biology, Department of Molecular Biosciences, University of Texas at Austin, 2506 Speedway Stop A5000, Austin, TX 78712 USA.; Institute for Cellular and Molecular Biology, Department of Molecular Biosciences, University of Texas at Austin, 2506 Speedway Stop A5000, Austin, TX 78712 USA.; The Department of Biochemistry & Microbiology, University of Victoria, Victoria, BC V8W 3P6, Canada.

**Keywords:** DNA repair, prolyl isomerase, homologous recombination, FKBP, olaparib, réparation d’ADN, prolyl isomérase, recombinaison homologue, FKBP, olaparib

## Abstract

FK506-binding proteins (FKBPs) alter the conformation of proteins via *cis-trans* isomerization of prolyl-peptide bonds. While this activity can be demonstrated in vitro, the intractability of detecting prolyl isomerization events in cells has limited our understanding of the biological processes regulated by FKBPs. Here we report that FKBP25 is an active participant in the repair of DNA double-strand breaks (DSBs). FKBP25 influences DSB repair pathway choice by promoting homologous recombination (HR) and suppressing single-strand annealing (SSA). Consistent with this observation, cells depleted of FKBP25 form fewer Rad51 repair foci in response to etoposide and ionizing radiation, and they are reliant on the SSA repair factor Rad52 for viability. We find that FKBP25’s catalytic activity is required for promoting DNA repair, which is the first description of a biological function for this enzyme activity. Consistent with the importance of the FKBP catalytic site in HR, rapamycin treatment also impairs homologous recombination, and this effect is at least in part independent of mTor. Taken together these results identify FKBP25 as a component of the DNA DSB repair pathway.

## Résumé

Les protéines de liaison du FK506 (FKBP) modifient la conformation des protéines au moyen de l’isomérisation *cis-trans* des liens peptidiques de la proline. Alors que cette activité peut être démontrée *in vitro*, la grande difficulté à détecter les manifestations de l’isomérisation de la proline dans les cellules a limité notre compréhension des processus biologiques régulés par les FKBP. Les auteurs rapportent ici que la FKBP25 est un participant actif dans la réparation des bris double brin (BDB) d’ADN. La FKBP25 influence le choix de la voie de réparation des BDB en favorisant la recombinaison homologue (RH) et en supprimant la renaturation simple brin. En accord avec cette observation, les cellules dépourvues de FKBP25 forment moins de foyers de réparation comportant Rad51 en réponse à l’étoposide et à la radiation ionisante, et elles dépendent du facteur de réparation-recombinaison Rad52 pour assurer leur viabilité. Ils ont trouvé que l’activité catalytique de FKBP25 est requise pour favoriser la réparation d’ADN, ce qui constitue la première description de la fonction biologique de l’activité de cette enzyme. En accord avec l’importance du site catalytique de FKBP dans la RH, le traitement à la rapamycine diminue aussi la recombinaison homologue et cet effet est au moins partiellement indépendant de mTor. Dans leur ensemble, ces résultats identifient la FKBP25 comme élément de la voie de réparation des BDB de l’ADN. [Traduit par la Rédaction]

## Introduction

FK506-binding proteins (FKBPs) are enzymes that catalyze the *cis-trans* isomerization of prolyl-peptide bonds to regulate substrate protein structure and function. Fifteen FKBPs are found in the human proteome and family members populate all cellular compartments, including the nucleus (Bonner and Boulianne 2017; Ghartey-Kwansah et al. 2018). In limited cases, FKBPs have been shown to enzymatically target chromatin-associated proteins (Dilworth et al. 2012; Hanes 2015), but the biological processes controlled by nuclear FKBPs are generally poorly understood.

FKBP25 is cytoplasmic/nuclear enzyme that directly binds both DNA (Prakash et al. 2016) and double-stranded RNA (Dilworth et al. 2017). We previously used affinity purifications and BioID proximity labeling to identify FKBP25-associated proteins; these include ribosomal proteins, RNA-binding proteins, elements of the cytoskeleton and chromatin-associated factors (Gudavicius et al. 2014; Dilworth et al. 2017). Included in these interactors were a number of regulators of the DNA damage repair process, including: histone H1, Ku86, Ku70, MDC1, DNA-dependent protein kinase (DNA-PKcs), Kap1, Parp-1, topoisomerase enzymes, nucleophosmin, and nucleolin (Dilworth et al. 2017). Therefore, the FKBP25 interactome suggests that FKBP25 may play a role in regulating DNA repair.

A collection of pathways share the task of repairing DNA lesions to minimize the accumulation of mutations, maintain genome instability, and prevent tumorigenesis (Jackson and Bartek 2009; Ciccia and Elledge 2010; Hanahan and Weinberg 2011). Of the types of lesions that occur, double-strand breaks (DSB), which completely sever the DNA fiber, are the most severe. Pathways that respond to these lesions, collectively termed the DNA Damage Response (DDR), have long been exploited in the chemotherapeutic treatment of cancer and researchers are now looking to the DDR in the development of precision therapeutics, particularly in tumors with impaired repair pathways. The development and use of PARP inhibitors to treat homologous recombination (HR)-defective breast and ovarian tumors highlights this concept (reviewed in O’Connor 2015). Therefore, identifying novel proteins and mechanisms involved in DNA repair is essential not only for our fundamental understanding of these pathways but this knowledge may elicit new candidates for targeted cancer interventions. Here we show that the prolyl isomerase FKBP25 is a participant in the repair of DNA double-stranded breaks. We find that FKBP25 influences DSB repair outcome downstream of DNA end resection by HR and suppressing the mutagenic single-strand annealing (SSA) pathway. This impact is in part mediated by FKBP25’s prolyl isomerase active site and is the first description of a biological role for this enzyme activity. We also provide evidence that inhibition of FKBP25 in combination with targeting of PARP may be beneficial in disrupting the DDR as a therapeutic strategy in HR-proficient tumors.

## Materials and methods

### Cell culture

U2OS cells (ATCC), U2OS DSB repair pathway reporter cells [DR-green fluorescence protein (GFP), SA-GFP, EJ5-GFP, and EJ2-GFP were a gift from Dr. Jeremy Stark, City of Hope, Duarte, California, USA] were cultured in Dulbecco’s modified Eagle medium (DMEM) containing 10% (*v*/*v*) fetal bovine serum (FBS; Sigma) and antibiotics (10 U/mL penicillin and 10 μg/mL streptomycin; ThermoFisher) at 37 °C and 5% CO_2_. Stable U2OS shRNA knockdown cells were generated by transfection with HuSH 29-mer shRNA expression vectors (Origene) using GenJet U2OS Transfection Reagent (SignaGen) followed by selection with puromycin (InvivoGen) 48 h post-transfection until colonies formed. Colonies were then isolated, expanded, and screened for knockdown by Western blot and RT-qPCR. Knockdown efficiency of siRNA and shRNA targeting sequences have been previously characterized and their sequences made available (Dilworth et al. 2018).

### Statistics

All numerical results reported are the mean ± SEM. The number of replicates, technical or biological, are indicated in corresponding figure legends. If not indicated otherwise in figure legends, 2-tailed Student’s *t* test for unpaired data was used to evaluate single comparisons between different experimental groups.

### Plasmids

FKBP25 expression plasmids were generated by subcloning a synthesized FKBP25 gene (GenScript) into a modified pcDNA5/FRT/TO vector (ThermoFisher). For the DSB repair pathway reporter assay, the I-SceI expression vector was used for generating site-specific double-strand breaks (Addgene # 26477; a gift from Dr. Maria Jasin, Memorial Sloan-Kettering Cancer Center, New York, N.Y., USA) and a pIRES2-DsRed-Express was used as a transfection control (Clontech — a kind gift from Dr. Bob Chow, University of Victoria, British Columbia, Canada). FKBP25-GFP and FKBP25-NLS-GFP were generated by subcloning from pcDNA 5 FRT vectors into pGFP-N1 (Clonetech) using standard molecular biology techniques.

### DSB repair pathway reporter assay

Assays were performed as previously described (Gunn and Stark 2012). For siRNA knockdown, cells were reverse transfected with 10 nmol/L siRNA in 12 well plates using the jetPRIME (Polyplus transfection). Cells were then incubated for 24 h and split to 6-well plates and incubated for a further 24 h. Forty-eight hours after the initial siRNA transfection, cells were co-transfected with 0.8 μg of I-SceI expression plasmid, 0.4 μg dsRED expression vector, and 10 nmol/L siRNA using Lipofectamine 2000 following the manufacturer’s instructions. Cells were incubated with the transfection mix for 3 h, then washed in 1× phosphate-buffered saline (PBS), and fresh growth medium was added. For over-expression studies, cells were co-transfected with 0.8 μg I-SceI and 0.4 μg FKBP25 expression vector using Lipofectamine 2000, for 3 h as above. For chemical treatments, compounds were added to fresh media after a 3 h transfection with 0.8 μg I-SceI as described above. Three days following transfections, the cells were harvested by trypsinization by adding 200 μL 1× trypsin-EDTA (ThermoFisher) per well, incubating for 3–5 min at room temperature, dispersing cells with the addition 200 μL growth medium and collecting in Fluorescent Activated Cell Sorting (FACS) tubes. After harvesting the cells, 200 μL formaldehyde (10%) was added (1:2 ratio) to fix, and samples immediately vortexed for 2–3 s at medium speed. Samples were then analyzed by flow cytometry on a BD FACS Calibur within 4 h of harvesting.

### Immunofluorescence (IF)

IF was performed as previously described (Leung et al. 2014). Briefly, U2OS cells expressing shGFP or shFKBP25 were directly seeded on coverslips for overnight incubation. For Etopside damage, cells were treated with 100 μmol/L Etopside for 20 mins, and then washed and incubated for 2 h. For the ionizing radiation experiments, cells were treated with 5 Gy delivered by a Faxitron X-Ray machine. After IR, cells were incubated for 2 h. After the indicated treatment, cells were pre-extracted with cytoskeletal (CSK) buffer for 5 min on ice, fixed with 2% (*v*/*v*) formalin for 15 min at room temperature, and blocked with PBS containing 3% bovine serum albumin (BSA). After blocking, the cells were incubated with primary antibody overnight. After 3× PBS washes, the cells were incubated with secondary antibody for 1 h at room temperature. Primary antibody used was RAD51 (ab133534; Abcam). The secondary antibody used was Alex Fluor 488 goat anti-mouse IgG (Invitrogen). After slide preparation, imaging was processed and analyzed with the *Z*-stacked setting using the FV10-ASW3.1 software on a Fluoview 1000 confocal microscope (Olympus).

### Cell proliferation assays

MTT (3-[4,5-dimethylthiazole-2-yl]-2,5-diphenyltetrazolium bromide) assays were performed as previously described (van de Loosdrecht et al. 1994). Briefly, for knockdowns the cells were reverse-transfected with siRNA, incubated O/N, then trypsinized, counted by hemocytometer, and plated at densities ranging from 5000 to 15 000 cells per well of a 96-well plate. For FKBP and mTOR inhibition, cells were counted and plated at different densities in media containing drugs at the indicated concentrations. Plates were incubated for 72–96 h and 20 μL of 5 mg/mL thiazolyl blue tetrazolium (Sigma) was added to each well and incubated for 2.5–3 h at 37 °C. The medium was removed and 150 μL of DMSO was added per well to solubilize the precipitant. The plates were incubated for a further 15 min at room temperature with shaking, and samples were read at OD 595 nm and OD 630 nm, as a reference, on an absorbance microplate reader (BioTek).

### Laser microirradiation

FKBP25-NLS-GFP recruitment to laser microirradiation was performed as previously described (Gong et al. 2015). Briefly, cells were seeded on glass-bottom dishes (Willco Wells) and incubated overnight. The following day, the cells were transfected with an FKBP25-NLS-GFP construct using Fugene HD (Promega) and incubated for 24 h. The cells were then incubated in fresh medium for another 24 h in the presence of 10 μmol/L 5-bromo-2′-deoxyuridine (BrdU) at 37 °C. A 405 nm solid-state laser was used to generate BrdU-dependent DNA damage. Following damage, GFP fluorescence was monitored by live confocal fluorescent microscopy using an Olympus FV1000 microscope. For quantification, the fluorescent intensity of the damage site as well as undamaged control region with same size from the same cell were directly recorded by the FV10-ASW3.1 software in real-time.

### Western blotting

Western blotting was performed by resolving proteins by SDS-PAGE and transferring to nitrocellulose membranes in phosphate transfer buffer (50 mmol/L sodium phosphate buffer (pH 6.8), 15% EtOH). The membranes were incubated in 10% skim milk for 30 min to block, and probed in 1:2000 dilution of primary antibody to FKBP25 (epitope residues 201—224; GeneScript) in 1% milk TBS-T (1× TBS with 0.1% Tween 20) for 1 h at room temperature followed by 3 washes in TBS-T. The blots were incubated with IRdye 680RD anti-rabbit (Mendel Scientific) at 1:5000 for 1 h at room temperature in 1% milk TBS-T, followed by 3 washes in TBS-T and one wash in 1× TBS, and imaging on an Odyssey Clx imaging system (Li-Cor).

### RT-qPCR

RNA was isolated using the TRIzol reagent (ThermoFisher) and cDNA prepared using the High-Capacity cDNA Reverse Transcription kit (ThermoFisher). cDNA was diluted 1:200 and used as a template in reactions using 2× Maxima SYBR green master mix (ThermoFisher). Samples were analyzed on an MX3000P qPCR system (Agilent Technologies) and fold-change calculated by the ΔΔ*C*_T_ method.

## Results

### FKBP25 influences DSB repair pathway usage

We previously used complementary immunoprecipitation mass spectrometry (IP-MS) and proximity labeling proteomic approaches to annotate FKBP25’s interacting proteins (Gudavicius et al. 2014; Dilworth et al. 2017). In addition to ribosomal proteins, RNA-binding proteins, and elements of the cytoskeleton, these experiments identified a number of chromatin-associated proteins including regulators of the DNA damage repair process, including: histone H1, Ku86, Ku70, MDC1, DNA-dependent protein kinase (DNA-PKcs), Kap1, Parp-1, topoisomerase enzymes, nucleophosmin, and nucleolin. These observations prompted us to investigate whether FKBP25 participates in the repair of DNA.

**Fig. 1.**
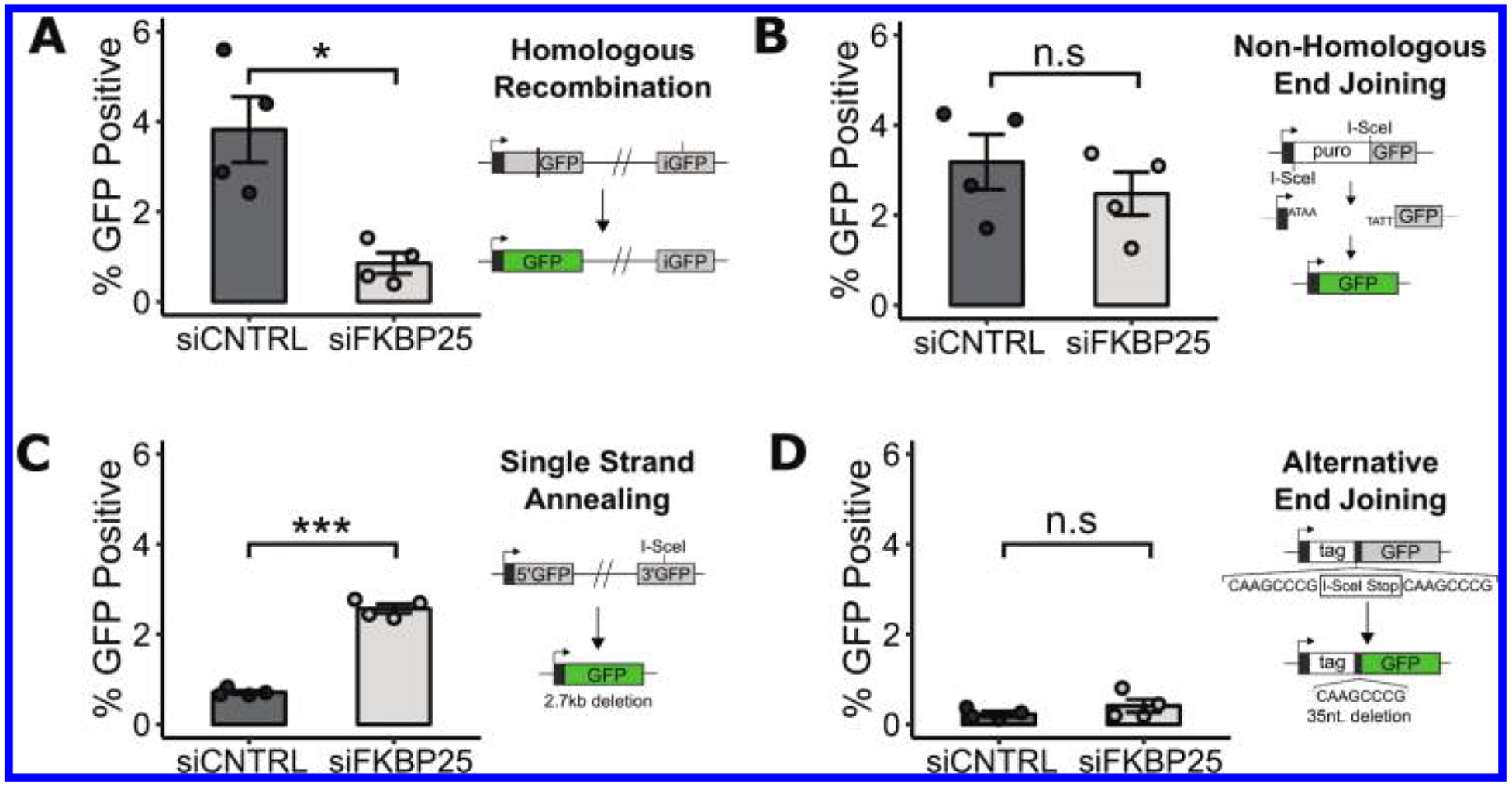
FKBP25 promotes homologous recombination and suppresses single-strand annealing double strand break (DSB) repair pathways. (A–D) Flow cytometry reporter assay measuring DSB repair pathway utilization in FKBP25 knockdown U2OS cells containing stably integrated reporters for (A) Homologous Recombination, (B) Non-Homologous End Joining, (C) single-strand annealing, and (D) Alternative End Joining. Error bars represent the standard error of 4 independent replicates. *, *P* < 0.05; ***, *P* < 0.001.

The repair of DNA DSBs normally proceeds through one of several pathways (Chapman et al. 2012). To determine whether FKBP25 participates in any of these repair pathways, we utilized a panel of four U2OS cell lines that each harbor a GFP-based reporter for successful repair of an induced lesions by classical non-homologous end-joining (c-NHEJ), homologous recombination (HR), single-strand annealing (SSA), or alternative end-joining (Alt-EJ) (Gunn and Stark 2012). We depleted FKBP25, using previously characterized siRNA reagents (Dilworth et al. 2018), in each reporter cell line and measured the pathway activity by flow cytometry. To normalize for transfection efficiency under knockdown conditions, a dsRed reporter was also co-transfected. We found that depletion of FKBP25 significantly impairs homologous recombination and promotes the error prone single-strand annealing pathway (Fig. 1). FKBP25 depletion did not significantly alter end-joining repair by either c-NHEJ or Alt-EJ. The decision between HR and SSA repair events occurs downstream of DNA-end resection by CtIP. For HR to proceed, Rad51 must displace RPA to form the presynaptic complex, a process critical for strand invasion (Krejci et al. 2012). Rad52, a mediator of SSA, can suppress RPA turnover and Rad51 loading onto ssDNA, in turn promoting repair by SSA (Rothenberg et al. 2008; Gibb et al. 2014). Therefore, the observation that FKBP25-depleted cells have reduced HR, and increased SSA, suggests that FKBP25 influences repair decisions downstream of DNA-end resection.

**Fig. 2.**
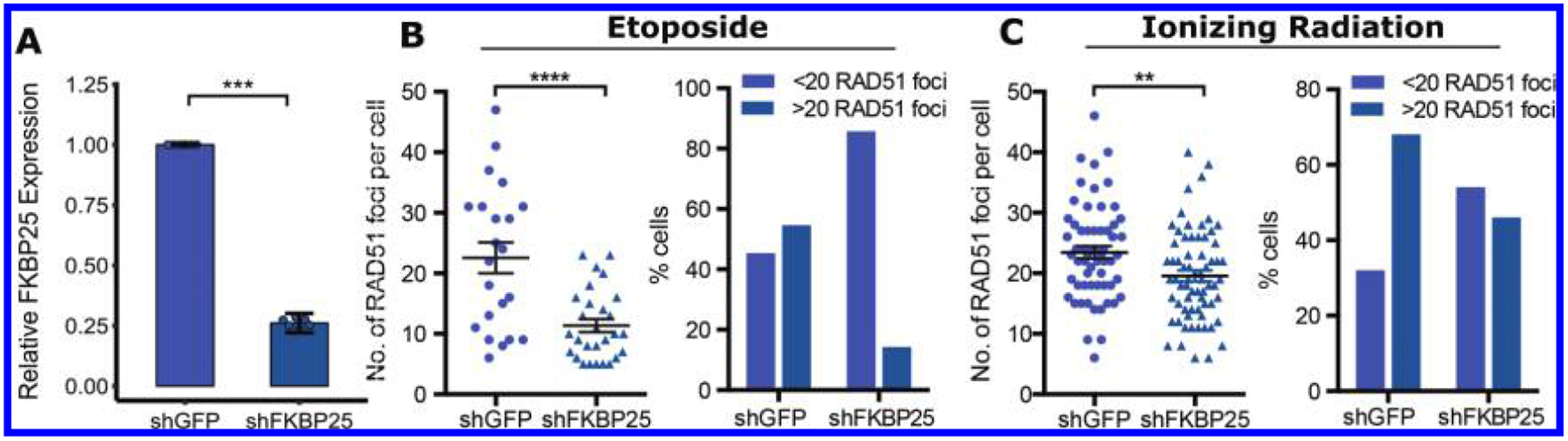
FKBP25 promotes Rad51 foci formation in response to DNA damage. (A) FKBP25 expression levels relative to GAPDH in U2OS shRNA knockdown cells. (B) Rad51 foci formation in response to etoposide. shRNA knockdown cells treated with 100 μmol/L washed and incubated for 2 h; 20 cells counted per sample. Also shown, quantification of cells with greater or less than 20 Rad51 foci per cell (C) Rad51 requirement in response to ionizing radiation. Cells treated with 5 Gy of radiation and incubated for 2 h; 60 cells counted per condition. Also shown, quantification of cells with greater or less than 20 Rad51 foci per cell. **, *P* < 0.01; ***, *P* < 0.001; ****, *P* < 0.0001.

**Fig. 3.**
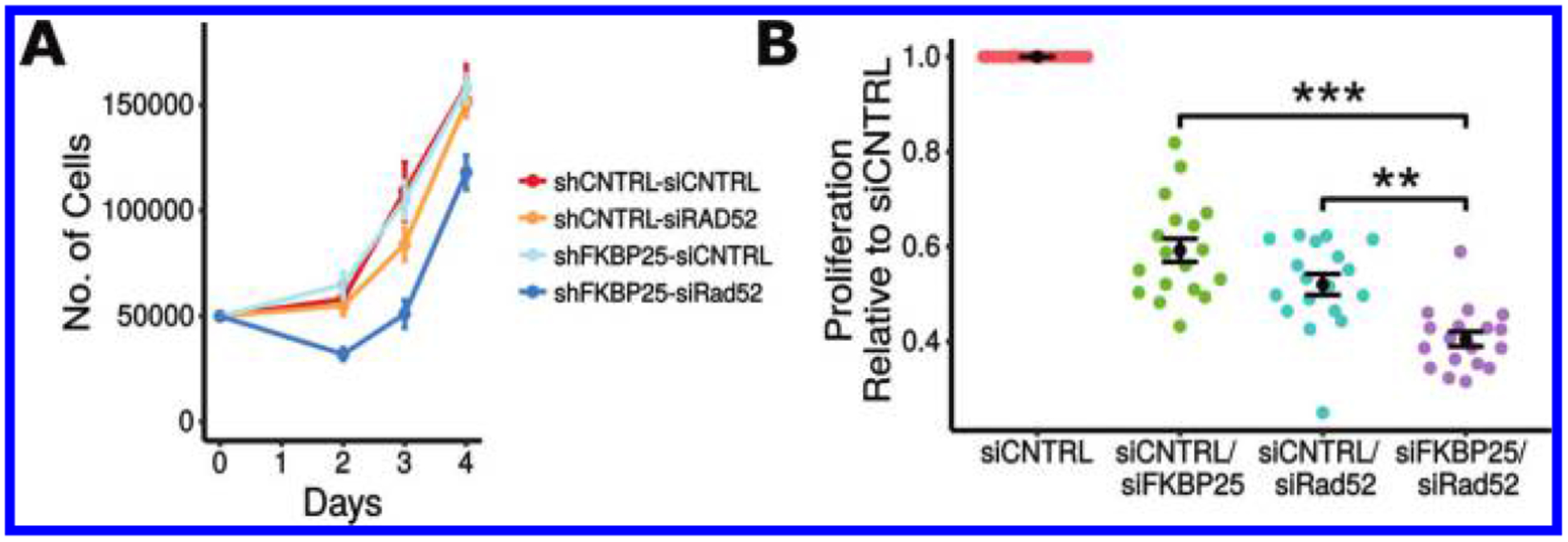
Cells deficient for FKBP25 and the single-strand annealing (SSA) repair factor Rad52 exhibit synthetic loss of viability. (A) Cell counting assay quantifying proliferation in U2OS cells stably expressing shRNA targeting green fluorescent protein (GFP) or FKBP25 and transfected with pooled siRNA targeting Rad52 or a non-targeting control. Error bars represent the standard error of 4 replicates in 2 independent experiments. (B) MTT proliferation assay of U2OS cells transfected with different combinations of siRNA, targeting FKBP25, Rad52, or a non-targeting control. Assay performed 72 h post-transfection. Data points shown are multiple measurements taken from 2 independent experiments. Error bars represent the standard error of 4 measurements across 4 independent transfections. **, *P* < 0.01; ***, *P* < 0.001.

To test this idea and further validate these results, we scored Rad51 foci formation in response to the DNA damaging agents etoposide or ionizing radiation (Fig. 2). Relative to control cells that express non-targeting shRNA, FKBP25-depleted cells display reduced Rad51 foci formation in response to either DNA-damaging agent. We note that cells with a robust response to either agent (>20 RAD51 foci per cell) are most affected by depletion of FKBP25. Because the shRNA targeting sequence used here is different from the siRNA used in the reporter cell line experiments, the observed repair deficit is unlikely to be the result of RNA interference off-target effects. We predicted that if cells have switched from HR-mediated repair to SSA when FKBP25 is depleted, they would become reliant on SSA for DSB repair and survival. To test this, we depleted Rad52, using a pooled siRNA approach, in combination with FKBP25 knockdown and evaluated viability by cell counts and the MTT proliferation assay. In support of our hypothesis, we find a synthetic-loss of viability relationship between FKBP25 and Rad52 (Fig. 3). Collectively, these results point to a role for FKBP25 in suppressing SSA to promote repair by homologous recombination.

### Mobilization of FKBP25 from laser microirradiation induced DSBs

**Fig. 4.**
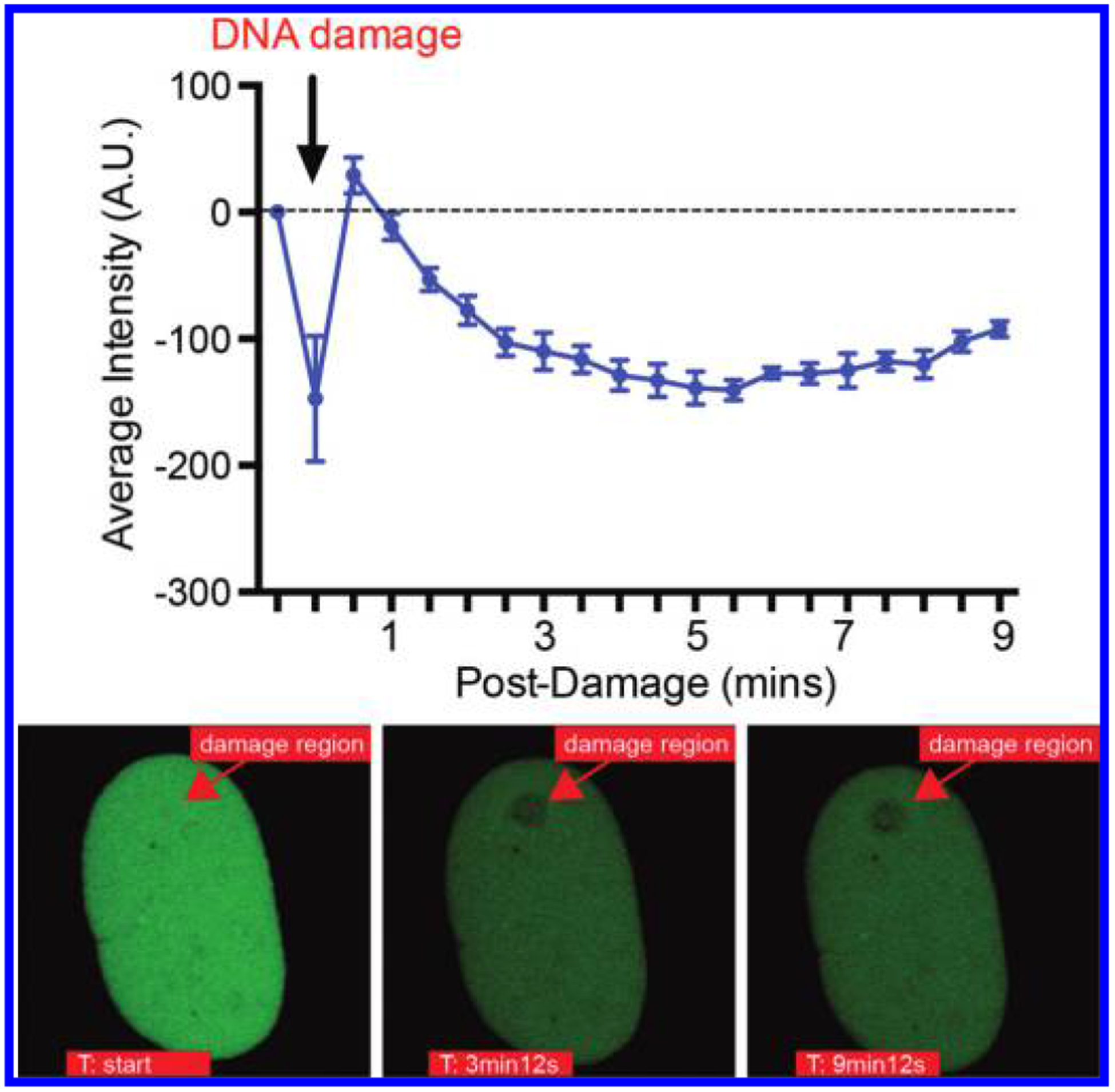
FKBP25 is displaced from laser microirradiation-induced DNA double-strand breaks. Quantification of fluorescent intensity of FKBP25-NLS-GFP after laser-induced damage (top). Images depicting the site of exclusion from laser-induced damage are shown below. The laser microirradiated area is indicated with an arrow.

FKBP25 interacts with DNA (Prakash et al. 2016) as well as core histones, linker histones, and RNA (Dilworth et al. 2017). As chromatin is actively reorganized around sites of damage to facilitate repair, we next determined whether FKBP25 is actively recruited to DNA double-strand breaks. We evaluated co-localization of an mCherry-LacI-FokI fusion and FKBP25-GFP at an induced DNA break near an integrated lacO array (Shanbhag and Greenberg 2013) and did not observe colocalization of FKBP25 at DSBs (data not shown). We next tested whether FKBP25 might visit DNA lesions transiently. To this end, we used live-cell imaging to study the localization of FKBP25-GFP to laser microirradiation induced DNA damage, which is a sensitive technique that captures kinetic information of protein localization to sites of DNA damage. Surprisingly, rather than accumulating at sites of DNA damage, FKBP25 appeared to be excluded from damaged chromatin within minutes post-damage (Fig. 4). It is noteworthy that a similar phenomenon has been observed for histone H1 as well as the heterochromatin-associated protein KAP-1; two FKBP25 interacting partners that are transiently displaced from IR-damaged chromatin in a PARP-1/ATM dependent manner (Guo et al. 1999; White et al. 2012; Strickfaden et al. 2016). It has recently become apparent that tight control over the chromatin environment surrounding DSBs is required for repair as during the DDR there is an initial expansion of chromatin followed by a phase of chromatin condensation (Burgess et al. 2014; Khurana et al. 2014; Li et al. 2014). These results suggest that FKBP25 displacement at sites of DSB may play a role in the reorganization of the chromatin environment to promote repair by homologous recombination.

### FKBP25’s catalytic activity is required to promote HR

**Fig. 5.**
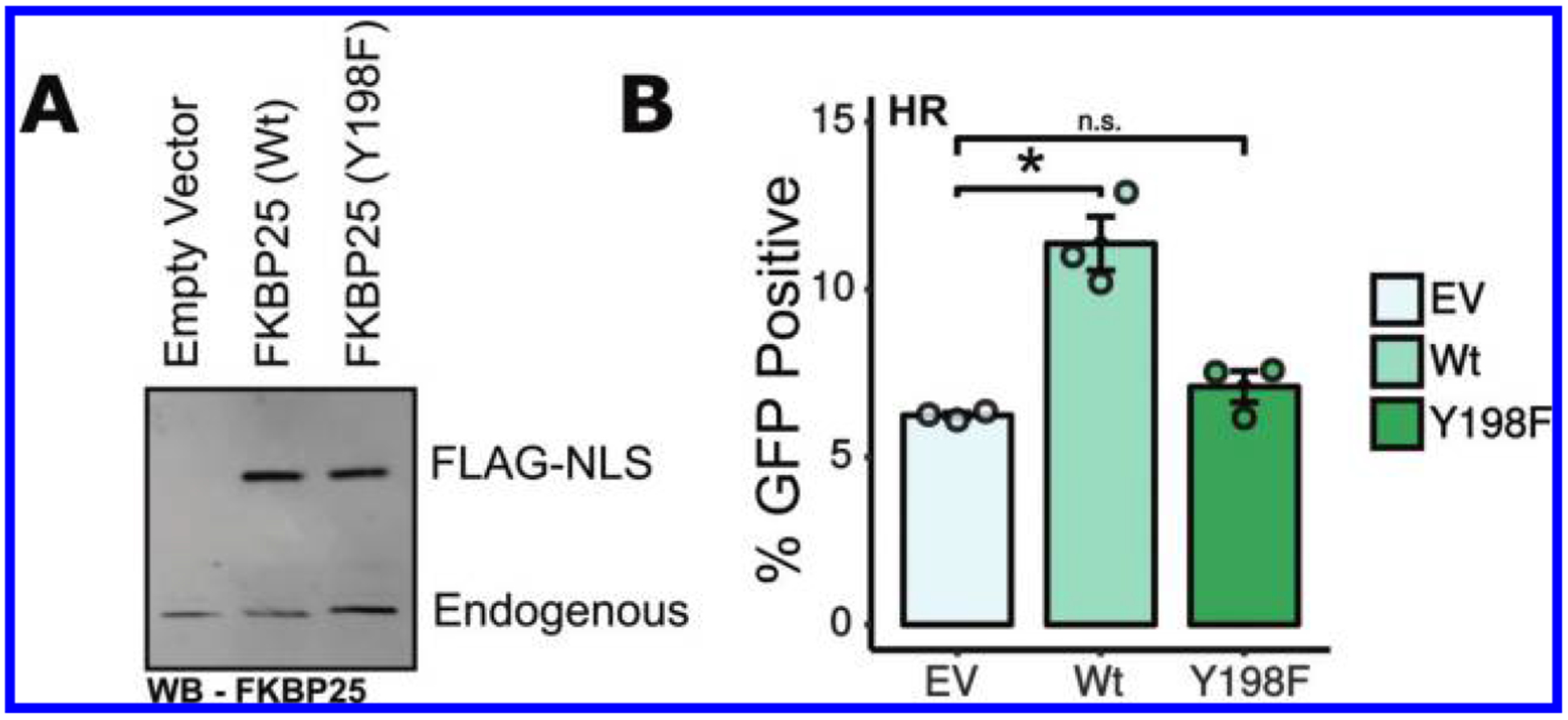
FKBP25’s catalytic activity is required for homologous recombination (HR). (A) Western blot analysis of the U2OS DR-GFP homologous recombination reporter cells transfected with I-SceI and either an empty vector control, FKBP25(Wt), or FKBP25(Y198F) expression vectors. (B) Quantification of GFP U2OS DR-GFP reporter cells transfected as in A by flow cytometry. Error bars represent standard error of 3 independent transfections. *, *P* < 0.05.

In addition to a recently described double-stranded RNA (Dilworth et al. 2017) and DNA (Prakash et al. 2016) binding activity, the FKBP25 protein contains a name sake FKBP prolyl isomerization domain. To determine whether this enzyme activity is involved in DNA repair processes, we expressed wildtype and catalytically inert (Y198F) (Gudavicius et al. 2013) forms of FKBP25 in cells used for the HR DSB reporter assay shown in Fig. 1A. While over-expressed FKBP25 significantly increased repair by homologous recombination, the catalytic-inert point mutant showed no difference from cells transfected with an empty vector control plasmid (Fig. 5). This indicates FKBP25’s catalytic activity likely promotes repair by HR. To our knowledge this is the first putative function for the prolyl isomerase action of FKBP25.

### Chemical inhibition of FKBPs disrupts HR

**Fig. 6.**
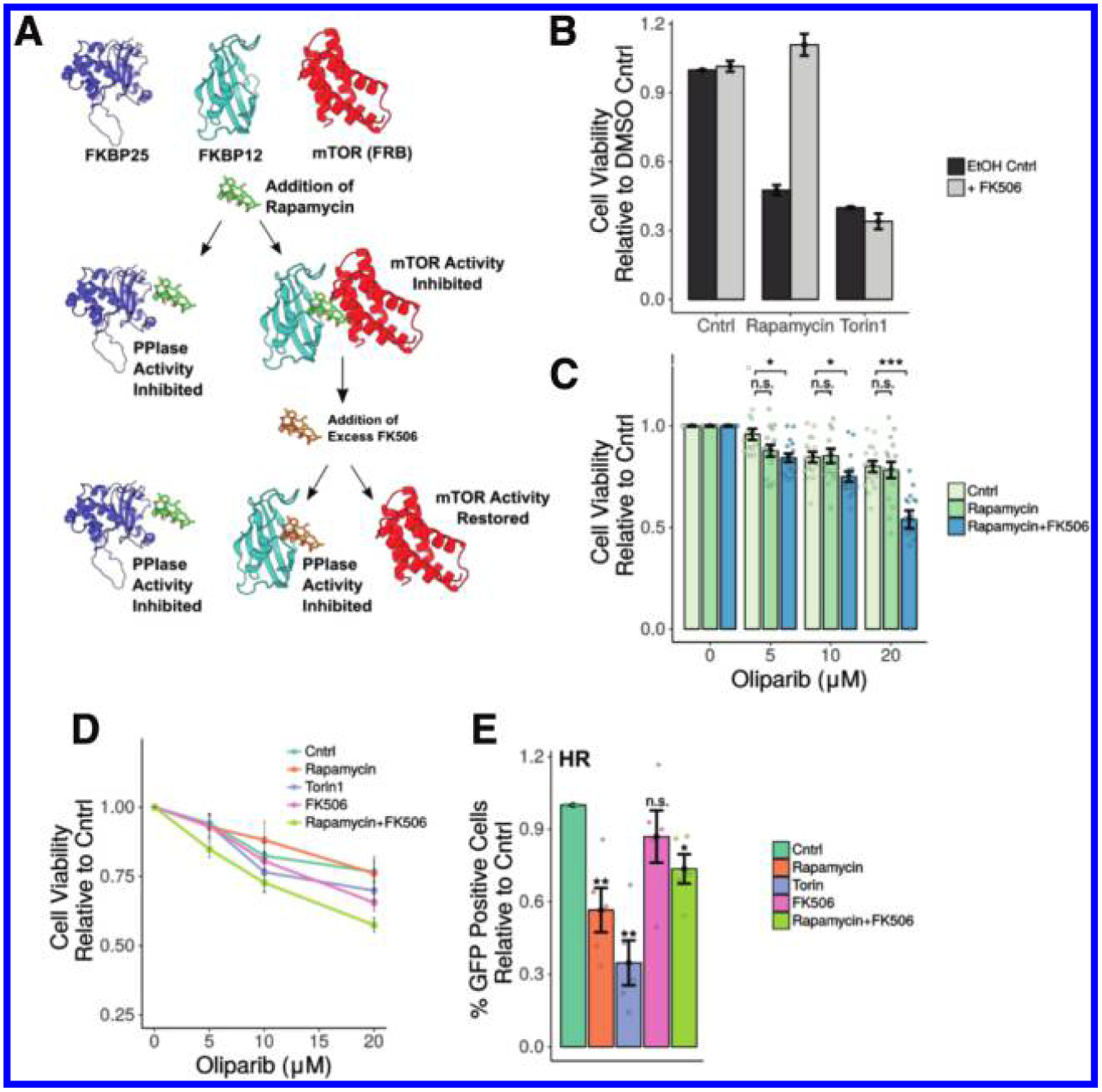
Inhibition of FKBPs impairs homologous recombination independently of mTOR. (A) A schematic presenting the strategy for chemical inhibition of FKBP25 by rapamycin without inhibiting mTOR. mTOR activity is restored by competition with FK506, which has a similar affinity for FKBP12 as rapamycin. [The following Protein Databank (PDB) entries were used in the generation of this figure; PDB ID 3FAP (Liang et al. 1999) and PDB ID 2MPH (Prakash et al. 2016)] (B) MTT proliferation assay as a proxy for the restoration of mTOR activity measuring proliferation. Cells were treated with either a dimethyl sulfoxide (DMSO) control, 10 nmol/L rapamycin, or 10 nmol/L Torin1 in the absence or presence of 2 μmol/L FK506. Error bars represent the standard error of 4 measurements across 4 independent experiments. (C) MTT proliferation assay of cells treated with increasing doses of the Parp-inhibitor olaparib in combination with 10 nmol/L rapamycin or 10 nmol/L rapamycin and 2 μmol/L FK506. Error bars represent the standard error of 4 measurements across 4 independent experiments. (D) MTT proliferation assay, cells treated as in (E). Error bars represent the standard error of 4 measurements from 2 independent experiments. (E) Flow cytometry reporter assay measuring the homolous recombinant double strand break repair pathway utilization in cells treated with DMSO control; 10 nmol/L rapamycin, 10 nmol/L Torin 1, and 2 μmol/L FK506; or 10 nmol/L rapamycin and 2 μmol/L FK506. Error bars represent standard error of 4 independent experiments. Significance relative to the DMSO control treated cells is shown above each bar. *, *P* < 0.05; **, *P* < 0.01.

Therapeutic strategies targeting the DDR are now being used successfully in the treatment of cancer (O’Connor 2015). For example, the PARP inhibitor olaparib has been shown to be successful as a maintenance treatment in recurrent hereditary BRCA-associated cancers, extending progression-free survival of patients (Kaufman et al. 2015). Given that FKBP25 regulates HR, we tested whether pharmacological inhibition of FKBP25 could sensitize cells to PARP inhibition. In contrast to most FKBPs, FKBP25 has a strong binding preference for rapamycin (*K*_i_ = 0.9 nmol/L) relative to FK506 (*K*_i_ = 200 nmol/L) (Dejmek et al. 2009). Unfortunately, assessing FKBP-mediated effects of rapamycin is convoluted by the fact that upon rapamycin treatment several FKBPs, including FKBP25, form a drug-induced heteromeric complex with mTOR that allosterically inhibits its kinase activity (Chen et al. 1995; Galat 2013). However, it is possible to overcome mTor inhibition of rapamycin by treating cells with excess FK506, which does not inhibit mTOR — this dual treatment maintains inhibition of FKBP25’s catalytic activity (März et al. 2013) (Fig. 6A). We adopted this strategy to dissect the involvement of FKBPs in HR and sensitization of cells to PARP inhibition. Indeed, excess FK506 rescues a rapamycin proliferative phenotype, but not that of the mTOR catalytic inhibitor Torin1 (Fig. 6B). This confirms that mTOR-independent rapamycin phenotypes can be explored in this way. Next, we examined sensitivity to PARP inhibition in the presence of rapamycin ± FK506 (Fig. 6C). In contrast to previous studies, we detected only modest effects of rapamycin treatment on the sensitivity to PARP inhibition (Peng et al. 2014). Because the assay used measures proliferation, without differentiating between cell death and senescence, we may not be observing the previously described synergy between treatments because proliferation is already greatly reduced in rapamycin-treated cells. When cells were co-treated with rapamycin and FK506, we observe that increasing doses of the PARP inhibition by olaparib impart a synergistic effect on proliferation (Fig. 6C). This indicates that inhibition of FKBP prolyl isomerase activity may provide a therapeutic route for potentiating PARP inhibitor activity in BRCA proficient cancer cells. Repeating this assay including the direct mTOR inhibitor Torin1 and FK506 treatment alone showed that the rapamycin-FK506 combination shows the greatest decrease in proliferation with increasing concentrations of olaparib (Fig. 6D). However, because Torin1 alone also sensitizes cells to olaparib, the mechanism at play is at least in part mTOR-dependent, as has been previously suggested (Mo et al. 2016). To evaluate whether this phenotype is the result of impaired HR, we monitored HR, via GFP reporter assay, in the presence of mTOR and FKBP inhibitors. In agreement with the previous literature (Chen et al. 2011; Mo et al. 2016), mTOR inhibition impairs HR (Fig. 6E). However, we also observed a reduction in HR with the rapamycin-FK506 combination, suggesting that FKBP inhibition, most likely through FKBP25, contributes at least in part to the synergistic mechanism described for PARP sensitization by rapamycin.

## Discussion

Here we expand on prior observations that FKBP25 physically interacts with a number of DSB repair factors by demonstrating that this prolyl isomerase promotes homologous recombination in a mechanism that likely involves catalytic activity. In the absence of FKBP25, there is a significant increase in repair by the error-prone SSA pathway. SSA can result in large deletions of the intervening sequence between stretches of homologous DNA, leading to genetic instability. Therefore, these results implicate FKBP25 in maintaining the integrity of the genome in cycling cells.

A multitude of DSB repair factors are actively recruited to DNA lesions, but because the repair process is dynamic, the removal of resident chromatin factors is also critical. We find that FKBP25 is displaced from sites of induced DNA damage. This behavior is notably similar to at least two of its interacting partners: KAP-1 and histone H1. KAP-1 establishes heterochromatin environments by recruiting repressive histone methyltransferase enzymes and heterochromatin protein 1 (HP1) (Iyengar and Farnham 2011). In response to DSBs KAP-1 is phosphorylated by ATM, triggering its diffusion into the nucleus, the relaxation of heterochromatin domains, and improved access for DNA repair (White et al. 2012; Geuting et al. 2013). Surprisingly, depletion of KAP-1 disrupts recruitment of Rad51 within euchromatin and heterochromatin (Baldeyron et al. 2011) and up-regulates alternative error-prone repair pathways (Geuting et al. 2013). These observations indicate that KAP-1 also plays an active role in DSB repair. Indeed, the KAP-1-HP1-SUV39H1 complex is recruited to DSBs, resulting in H3K9me3-dependent activation of ATM (Ayrapetov et al. 2014). Thus, the eviction of heterochromatin proteins occurs only transiently, suggesting that dynamic cycles of relaxation and compaction of the chromatin template is vital for repair by HR. The same also seems to be true for the linker histone H1, which had the highest enrichment in our FKBP25 BioID proteomic screen (Dilworth et al. 2017). There is also a significant overlap in the protein-protein interactions of FKBP25 and histone H1 (Szerlong et al. 2015). Like KAP-1, histone H1 is initially evicted from chromatin in response to DSBs (Strickfaden et al. 2016) and its presence can prevent Rad51 loading (Machida et al. 2014). However, it is also important at break sites, initiating and amplifying ubiquitination signaling, which is required for the recruitment of downstream repair proteins (Thorslund et al. 2015). Interestingly, histone H1 has been shown to be a target for Pin1-mediated prolyl isomerization, with implications for its binding to chromatin (Raghuram et al. 2013). Given these findings, histone H1 may be a target for prolyl isomerization by FKBP25 in the regulation of DNA repair. If true, this would imply that FKBP25’s catalytic activity might promote HR through remodeling the chromatin environment, which would be similar to the histone-binding/chromatin targets and recombination checkpoint functions of the orthologous nuclear FKBPs in yeast (Hochwagen et al. 2005; Nelson et al. 2006; Macqueen and Roeder 2009; Monneau et al. 2013; Ohkuni et al. 2014; Edlich-Muth et al. 2015; Leung et al. 2017). While further work is required, a role in DNA damage repair may explain FKBP25’s, so far inexplicable, association with chromatin.

Alternatively, FKBP25 regulation of DNA damage may come as result of its regulation of microtubule dynamics (Dilworth et al. 2018). Microtubule-dependent transport of the DNA damage repair proteins DNA-PK, NBS1, MRE11, and 53BP1 promotes their nuclear localization (Poruchynsky et al. 2015). While a mechanistic connection between FKBP25 stabilization of microtubules (Dilworth et al. 2018) and roles in HR cannot be directly ruled out, we note that FKBP25 catalytic activity impacts DNA repair but not microtubule polymerization (Dilworth et al. 2018). This, coupled with the fact FKBP25 is actively displaced from chromatin, suggests that FKBP25’s involvement in DNA repair is more likely a result of the nuclear chromatin-proximal, rather than cytoplasmic and microtubule-associated functions.

Rapamycin (Sirolimus) and FK506 (Tacrolimus) are well-characterized inhibitors of FKBPs that are primarily deployed to block mTor (Heitman et al. 1991) or calcineurin (Liu et al. 1991; Ho et al. 1996). These effects are mediated through a gain-of-function complexes with FKBP12, not blockage of prolyl isomerase activities. However, it is recognized that interaction of these small molecules with other FKBPs could mediate some of the effects of these drugs (März et al. 2013). Because rapamycin is known to suppress HR (Chen et al. 2011), we exploited FKBP25’s preference for rapamycin over FK506 to determine whether FKBP25 is likely to contribute to this effect. While targeting of FKBPs with rapamycin may work to potentiate the action of DDR targeted therapies, a significant drawback is the immunoinhibitory effects of this drug that are mediated by FKBP12-mTor. It is well-appreciated that the immune system plays a vital role in the body’s defense against cancer (Zitvogel et al. 2013). Thus, the development of FKBP inhibitory molecules that discriminate between FKBP catalytic pockets (Blackburn and Walkinshaw 2011) might hold the potential to suppress DNA repair via FKBP25, while not dampening the immune system via FKBP12-mTor. While there is still much to be learned, this study provides supporting evidence that inhibiting FKBP25 may be an effective route to enhance targeting of the DDR.
